# Immunotherapy in Advanced Biliary Tract Cancers

**DOI:** 10.3390/cancers13071569

**Published:** 2021-03-29

**Authors:** Alice Boilève, Marc Hilmi, Cristina Smolenschi, Michel Ducreux, Antoine Hollebecque, David Malka

**Affiliations:** 1 Département de Médecine Oncologique, Gustave Roussy, F-94805 Villejuif, France; alice.boileve@gustaveroussy.fr (A.B.); Cristina.SMOLENSCHI@gustaveroussy.fr (C.S.); michel.ducreux@gustaveroussy.fr (M.D.); antoine.hollebecque@gustaveroussy.fr (A.H.); 2 Université Paris-Saclay, F-91190 Saint-Aubin, France; 3 Département D’Innovations Thérapeutiques et D’Essais Précoces, Gustave Roussy, F-94805 Villejuif, France; marc.hilmi@gustaveroussy.fr

**Keywords:** biliary tract cancers, cholangiocarcinoma, immune checkpoint inhibitor, drug combination, immunotherapy, vaccine

## Abstract

**Simple Summary:**

A new era has emerged in oncology in the last ten years with the development of immune therapies. However, single-agent immune therapy such as immune checkpoint inhibitors seems to have a limited clinical activity in biliary tract cancers and are still largely investigational, except for the few patients with microsatellite-instable tumors. Here, we review: (i) the molecular and immune landscape of biliary tract cancers, (ii) the existing results of immune therapies in biliary tract cancers, and (iii) the future of immune therapies in biliary tract cancers, with the identification of predictive biomarkers for response to these therapies, and the ongoing therapeutic trials.

**Abstract:**

Biliary tract cancers are rare tumors with a poor prognosis. Two-thirds of these primary liver malignancies are diagnosed at advanced stages where therapeutic options are limited. Whereas several molecular targeted therapies emerge in biliary tract cancers, immunotherapy is still investigational, the only approved immunotherapy to date being the immune checkpoint inhibitor pembrolizumab for the small fraction of patients with microsatellite-instable tumors. In microsatellite-stable, pre-treated biliary tract cancers, single-agent immune checkpoint blockade has a limited albeit often long-lasting clinical activity in a still ill-defined subgroup of patients. The identification of predictive biomarkers will allow a better selection of patients that may benefit from immunotherapy. Combinations of immunotherapies with each other, with chemotherapy or targeted molecular therapies are being investigated in early lines of therapy, including first-line.

## 1. Introduction

Biliary tract cancers (BTC) are a heterogeneous group of uncommon epithelial tumors arising from the biliary duct cells. They represent the second most common primary liver malignancy after hepatocellular carcinoma, accounting for 15% of all primary liver tumors and 3% of gastrointestinal cancers [1,2]. Incidence amounts to some 10,000 new cases/year in Europe (0.5 to 3 cases per 100,000 people) and 12,000 new cases/year in the United States (1.6 cases per 100,000 people) [3,4]. Incidence is higher in Asia, with 5.7 to 85 cases per 100,000 people [5,6]. Based on anatomical location, BTCs are subdivided into: (i) intrahepatic cholangiocarcinoma, extrahepatic cholangiocarcinoma that comprises perihilar cholangiocarcinoma and distal cholangiocarcinoma, and gallbladder carcinoma [7]. This anatomical classification parallels with distinct biological and molecular features.

The incidence of BTCs is increasing, mainly for intrahepatic cholangiocarcinomas but also for extrahepatic cholangiocarcinomas [5], probably because of metabolic and infectious risk factors. The main described etiologic factors associated with cholangiocarcinomas are chronic viral infections (hepatitis virus B and hepatitis virus C), cirrhosis or nonalcoholic fatty liver disease, obesity, alcohol consumption, tobacco consumption, diabetes but also chronic inflammation of the biliary tract and bile stasis (mainly due to sclerosing cholangitis or liver fluke infections in endemic areas (Asia)) [8,9].

Surgery is the only potential curative treatment for BTCs, but approximately 70% of patients are diagnosed at advanced stages due to absence of specific symptoms [10,11]. Moreover, tumor relapse is frequent after curative-intent surgical resection [12,13,14]. Therapeutic options for non-resectable disease are scarce and treatment is only palliative [7]. Based on the results of the ABC-02 trial, the combination of cisplatin plus gemcitabine [CISGEM regimen] is the standard first-line treatment in this setting, with median overall survival (OS) and progression-free survival (PFS) in the CISGEM group of 11.7 and 8.0 months, respectively [15]. The proposed second-line chemotherapy is the combination of fluorouracil and oxaliplatin (FOLFOX regimen), based on the results of the ABC-06 trial—the only phase 3 trial reported to date in this setting—albeit efficacy results were modest (median OS, 6.2 vs. 5.3 months; overall response rate, ~5%) [16]. Beyond second line, there is no validated standard treatment.

In the last decade, immune therapies have greatly improved the treatment and outcomes of solid tumors, as illustrated with melanoma [17], lung cancers [18,19] or renal cancers [20]. Main targets are the immune checkpoints cytotoxic T lymphocyte antigen 4 (CTLA-4) and programmed cell death 1 (PD-1), receptors located on T-cells that regulate immune responses at the priming phase in lymph nodes and at the effector phase in the tumor, respectively [21]. Immune checkpoint inhibitors are mainly represented by monoclonal antibodies directed against CTLA-4 or PD-1 or its ligand (programmed cell death ligand-1, PD-L1), restoring the immune function of ‘exhausted’ T cells and depleting immunosuppressive regulatory T lymphocytes (Treg) [22]. While treatment landscape of several solid tumors has been deeply changed by novel immune therapies, their role is still unclear in advanced BTC [23] and combination of immune therapies could be valuable in BTC [24].

Here, we review: (i) the molecular and immune characterization of BTCs, paving the rationale for immune therapies in these tumors, (ii) the existing results and trials of immune therapies in BTCs, and (iii) the future of immune therapies in BTCs, with the identification of predictive biomarkers for response to these therapies and the ongoing therapeutic trials.

## 2. Molecular and Immune Characterization of Biliary Tract Cancers

### 2.1. Molecular Landscape of Biliary Tract Cancers

The heterogeneity and complexity of cholangiocarcinomas have been unraveled by next-generation sequencing. Besides cancers with prevalent oncogenic mutations (e.g., *BRAF* mutation in melanoma, *c-kit* or *PDGFR* mutations in gastrointestinal stromal tumors), BTCs are now known to present one of the highest frequencies of targetable molecular alterations across cancer types [25,26,27,28,29,30]. Notably, molecular patterns can be paralleled with the anatomical and histological classification of BTCs [31,32]. Isocitrate dehydrogenase gene (*IDH*) mutations and fibroblast growth factor receptor 2 (*FGFR2*) fusions are found almost exclusively in intrahepatic cholangiocarcinomas, with frequencies of approximately 15% and 20% respectively [33,34,35,36,37], whereas human epidermal growth factor receptor-2 gene (*HER2*) aberrations are observed in approximately 15% of cases of extrahepatic cholangiocarcinomas and gallbladder carcinomas [38]. Several targeted therapies have been or are currently tested in BTCs, leading notably to the FDA accelerated approval of pemigatinib, an oral and selective inhibitor of *FGFR1*, *2* and *3*, for the treatment of patients with previously treated, unresectable, locally advanced or metastatic cholangiocarcinoma that exhibits a *FGFR2* rearrangement or fusion [39]. Phase 3 results are also positive for ivosidenib (AG120), an oral inhibitor of *IDH1* in patients with *IDH1*-mutated cholangiocarcinoma who have failed one or two prior treatment lines [40].

### 2.2. Immune Microenvironment

**Figure 1:**
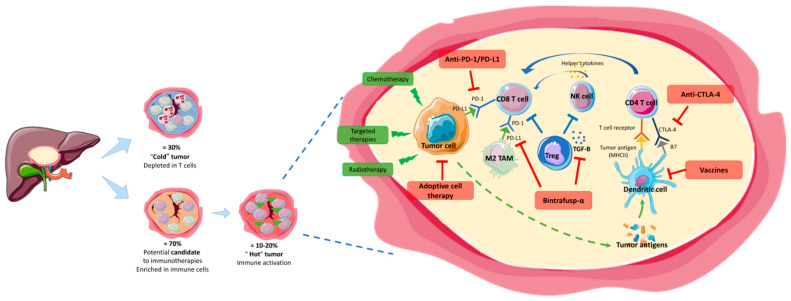
Biological rationale for use of immune therapies in BTC. Left panel: Approximately 70% of BTC are highly infiltrated in immune cells with a strong expression of the immune checkpoints such as PD-L1 in 10–20% of cases, whereas about 30% of BTC are depleted in cytotoxic lymphocytes (T cells). Right panel: Immune BTC microenvironment and potential therapeutic implications. CTLA-4: cytotoxic T-lymphocyte antigen-4; MHC: major histocompatibility complex; PD-1: programmed cell death 1; PD-L1: programmed cell death-ligand 1; TAM: Tumor-associated macrophages.

Although the role of the immune microenvironment in BTC progression and therapy resistance is well established [41], a better understanding of its specific functions is needed [42]. According to The Cancer Genome Atlas (TCGA) initiative, BTCs seem to be rich in immune cells in 70% of cases and depleted in lymphocytes in 30% with a balanced macrophage to lymphocyte ratio in most cases [43]. Immunohistochemistry studies showed that the type of immune infiltrate relates to different prognostic values. Prolonged survival is associated with CD8-positive tumor-infiltrating lymphocytes (TIL), natural killer lymphocytes, and major histocompatibility complex (MHC) class I expression [28,29,30]. At the opposite, M2-macrophages and neutrophils are associated with poor survival while Treg cells showed inconsistent prognostic value [44,45]. Furthermore, between 10% to 30% of BTCs express PD-L1 in tumor cells [46] and have a higher density of TIL, both of which are associated with a better response to immune checkpoint inhibitors [47] (Figure 1).

Molecular studies in BTCs identified a subgroup of tumors that may be good candidates for immune checkpoint inhibitors. Nakamura et al. [28] described four subtypes of BTC according to the gene expression of 260 tumors. Almost 40% of patients were classified in Cluster 4 with higher mutation load and higher expression of immune checkpoint genes (*LAG3, CTLA4, PDCD1, TNFRSF9, BTLA, IDO1, HAVCR2*, and *TNFRSF4*). This subgroup favorable to immune checkpoint inhibitors was also associated with a poor prognosis. Similarly, Jusakul et al. [25] defined four subtypes of BTC based on the liver fluke status. Cluster 3 included intrahepatic cholangiocarcinomas which were mostly fluke-negative and overexpressed immune checkpoint genes (*PD-1, PD-L2,* and *BTLA*). These immunogenic intrahepatic cholangiocarcinomas were mutually exclusive with IDH/FGFR-driven intrahepatic cholangiocarcinomas (Cluster 4).

In addition, between 5% and 10% of BTCs display DNA mismatch repair deficiency and/or microsatellite instability (MSI) [48]. This phenotype is characterized by a high load of neoantigens that activate antitumor T-cell response and has been associated with durable responses to immune checkpoint inhibitors in several solid tumors including BTCs [49]. Wardell et al. [50] analyzed 412 BTCs and found that 11% harbored deleterious germline mutations of cancer-predisposing genes increasing the tumor mutational burden (TMB) (*RAD51D, MLH1, MSH2, POLD1, POLE,* and *ATM*). Somatic or germline mutations in DNA mismatch repair genes (e.g., *MLH1, MSH2*) or DNA polymerases (e.g., *POLD1, POLE*) were displayed in all hypermutated tumors.

Overall, a subset of patients with BTCs are theoretically good candidates for immune checkpoint inhibitors based on their high TMB, level of expression of PD-L1 and TIL [51]. There is currently no immune checkpoint inhibitor development in selected subpopulations associated with an immune signature in BTCs. Translational studies on biological samples from ongoing trials are mandatory to correctly identify patients with BTCs sensitive to immune checkpoint inhibitors.

## 3. Current Results of Immune Therapies in Biliary Tract Cancers

### 3.1. Immune Checkpoint Inhibitor Monotherapy

Microsatellite instability (MSI) is observed in up to 10% of intrahepatic cholangiocarcinomas, 5–13% of extrahepatic cholangiocarcinomas and 5% of gallbladder carcinomas. Immune checkpoint inhibitors were shown to be highly active in patients with MSI tumors including some patients with BTCs, with objective response rate (ORR) of ~40% [52], making immune checkpoint inhibitors a good option for MSI BTCs.

Activity of immune checkpoint inhibitors was also tested in early studies in BTCs regardless of MSI status (Table 1). Safety and activity of the anti-PD1 monoclonal antibodies nivolumab was studied in a phase 2 study that included 54 refractory, microsatellite-stable BTC patients [53]. Overall, 10 out of 45 (22%) evaluable patients achieved a partial tumor response, with a disease control rate (DCR) of 60%. Median OS was 14.2 months (95% CI: 6.6-not reached) and median PFS was 4.0 months (95% CI: 2.3–6.0). Toxicity profile was favorable with 20% of grade 3/4 treatment-related adverse events. However, a lower efficacy of nivolumab alone was reported in a Japanese phase 1 study [54]. Among 30 patients with pre-treated BTCs, only one patient (with a MSI tumor) had an objective response. Median OS was 5.2 months (90% CI: 4.5–8.7) and median PFS was 1.4 months (90% CI: 1.4–1.4). Grade 3–4 treatment-related adverse events were reported by three (10%) patients (rash, maculopapular rash, and amylase increase).

In a phase 1b study (KEYNOTE-028) including pre-treated patients with PD-L1-positive BTC (≥1% PD-L1-positive cells), 24 patients received pembrolizumab monotherapy [56,57]. ORR was 13% (3/23, all partial responses) and median duration of response (DOR) was not reached (range, 21.5 to 53.2+ months). Median OS and PFS were 5.7 months (95% CI, 3.1‒9.8) and 1.8 months (95% CI, 1.4‒3.1), respectively, and 12-months OS rate was 27.6%. Overall, the safety profile was manageable with only 16.7% of patients with grade 3 treatment-related adverse events (no grade 4/5). In a phase 2 study (KEYNOTE-158) of pembrolizumab in pre-treated BTC patients, ORR was 5.8% (6/104 partial responses, 95% CI, 2.1%–12.1%) and median DOR was not reached (range, 6.2–26.6+ months) [57]. Median OS and PFS were 7.4 (95% CI: 5.5–9.6) and 2.0 (95% CI: 1.9–2.1) months, respectively. Among PD-L1-positive patients (*n* = 61) and PD-L1-negative patients (*n* = 34), ORR was 6.6% (4/61) and 2.9% (1/34), respectively. Grade 3 to 5 treatment-related adverse events were seen in 13.5% of patients (no grade 4; grade 5 renal failure, *n* = 1). None of the patients in these two studies had MSI tumors [57]. Complete response of extrahepatic cholangiocarcinoma or gallbladder carcinoma after pembrolizumab alone has been reported occasionally [72].

Durvalumab was also tested in 42 pre-treated Asian patients with BTC (59% PD-L1 ≥1%, 14% PD-L1 ≥ 25%) [58]. Grade ≥3 treatment-related adverse events occurred in 19% of patients. Two patients (4.8%) had a partial response and DCR at 12 weeks was 16.7%. Median DOR was 9.7 months. Median OS was 8.1 months (95% CI, 5.6–10.1) and median PFS was 2.0 months.

Bintrafusp alpha (M7824), a bifunctional fusion protein that targets PD-L1 and transforming growth factor beta (TGF-β), gave interesting results in 30 pre-treated BTC patients (53% PD-L1 > 1%) [59,60]. Ten patients (33%) experienced grade ≥3 treatment-related adverse events and three deaths due to adverse events were reported (1 death was due to septic shock, and two deaths due to interstitial lung disease). Seven patients (23.3%) had an objective response with long-lasting responses in 8 of 30 patients (27%). ORR was 25% in PD-L1-positive group and 15.4% in PD-L1-negative group. Median OS was 12.7 months (95% CI: 6.7–15.8). Recently, primary results from the Phase II INTR@PID BTC 047 study were released [61]. This study evaluated bintrafusp alpha as a monotherapy in the second-line treatment in 159 patients with locally advanced or metastatic BTC who have failed or were intolerant to first-line platinum-based chemotherapy. ORR was 10.1% (95% CI: 5.9% to 15.8%) per Response Evaluation Criteria in Solid Tumors (RECIST) criteria, version 1.1. More results should be available soon for this study.

Overall, all of these studies reported a favorable safety profile, albeit on a limited number of patients. Single-agent immune checkpoint inhibitors may benefit in a small, but important patient subset, in which long-lasting objective responses or disease stabilizations may be observed even after multiple prior treatment lines. Whether these encouraging results are obtained more frequently in PD-L1-positive patients needs to be confirmed, and results from phase 3 studies are pending (see Section 4.2).

### 3.2. Combinations with Immune Checkpoint Inhibitors

Several trials have studied combinations of immune checkpoint inhibitors with each other or with chemotherapy (Table 1). The abovementioned phase 1 study of single-agent durvalumab also tested durvalumab combined with tremelimumab (*n* = 65) [58]. Grade 3–5 adverse events occurred in 23% of patients (compared with 19% for durvalumab alone), with five patients who discontinued treatment for treatment-related adverse events, and one treatment-related death (drug-induced liver injury). ORR was 7.7% and DCR at 12 weeks was 32.2%. Median DOR was 8.5 months and median OS was 10.1 months (95% CI: 6.2–11.4). Durvalumab and tremelimumab were also tested in combination with CISGEM in a phase 2 study that included 121 BTC patients in first-line, 45 treated with CISGEM and durvalumab and 46 with CISGEM, durvalumab and tremelimumab (30 patients also received this quadruplet therapy in a biomarker cohort) [62]. Median ORR was 50.0%, 73.3% and 73.4% in the biomarker, triplet and quadruplet cohort, respectively, with DCR of 96.7%, 100% and 97.8% and median DOR of 11.0, 9.8, and 9.1 months. Median PFS was 13.0 months (95% CI: 10.1–15.9), 11.0 months (95% CI: 7.0–15.0) and 11.9 months (95% CI: 10.1–13.7), respectively, and median OS was 15.0 months (95% CI: 10.7–19.3), 18.1 months (95% CI: 11.3–24.9) and 20.7 months (95% CI: 13.8–27.6). The most frequent grade 3/4 adverse events were neutropenia (50.4%), anemia (35.5%) and thrombocytopenia (16.5%). The combination of CISGEM and durvalumab is currently investigated in the TOPAZ-1 phase 3 trial (ClinicalTrials.gov Identifier: NCT03875235). The combination of durvalumab and tremelimumab with weekly paclitaxel is currently investigated in the IMMUNOBIL PRODIGE 57 trial (ClinicalTrials.gov Identifier: NCT03704480). A safety run-in was performed after inclusion of 20 patients and led to discontinuation of the triplet arm (durvalumab, tremelimumab and paclitaxel) due to dose-limiting toxicities in six patients including an unexpected increase in anaphylactic adverse events [73]. The study continues as a single-arm phase 2 study of durvalumab and tremelimumab.

Nivolumab was also tested in combination with CISGEM in the abovementioned Japanese phase 1 study of single-agent nivolumab [54]. In the combined therapy cohort, the commonest treatment-related adverse events were neutropenia (grade 3–4 in 23 (77%) patients) and thrombocytopenia (grade 3–4 in 15 (50%) patients). Median OS and PFS were 15.4 (90% CI 11.8-not estimable) and 4.2 months (90% CI 2.8–5.6), respectively. Eleven of the 30 patients had an objective response (ORR 36.7%).

Toripalimab, an anti-PD-1 antibody, was combined to gemcitabine and the oral fluoropyrimidine S-1 in a phase 2 study of 39 patients with advanced BTCs [63]. Of 34 evaluable patients, ORR was 20.6% and DCR was 85.3%. Median PFS was 6.7 months (OS was immature). Grade 3–4 hematological and non-hematological adverse events were observed in 69.2% and 20.5% of patients, respectively.

Nivolumab or pembrolizumab were also tested in association with the oral, antiangiogenic, tyrosine kinase inhibitor lenvatinib in 32 patients with pre-treated intrahepatic cholangiocarcinomas [64,74,75]. ORR was 25% and DCR was 78.1%. Median PFS was 4.9 months (95% CI: 4.7–5.2 months) and median OS was 11.0 months (95% CI: 9.6–12.3 months). Grade 3 adverse events occurred in 59.3% of patients (one grade 4 adverse event).

Recently, results of the combination of durvalumab and tremelimumab with radiotherapy were presented [65]. The rationale is based on the observation of systemic tumor responses (abscopal effect) in metastatic, MSS pancreatic or colon cancer—i.e., tumors notoriously resistant to immune checkpoint inhibitors—when combining PD-1/CTLA-4 inhibitors with radiotherapy [76]. Fifteen pre-treated BTC patients were included and received durvalumab and tremelimumab along with radiotherapy to a single metastatic site (three fractions of 8 Gy at cycle 2 every other day). Dose-limiting toxicities occurred in 3 patients during the safety run-in, and three patients did not reach radiotherapy. DCR was 33% with a 17% partial response and 8% complete response for the patients who received radiotherapy. DOR was 26, 52, 122 and 254+ days for four patients with disease control. Grade 3-4 toxicities were observed in nine out of 15 patients (60%).

### 3.3. Other Types of Immunotherapy

#### 3.3.1. Vaccines

Other types of immune therapies have been tested in phase 1/2 studies, such as vaccines and cellular therapies. Vaccination studies have yielded modest results in advanced BTCs (Table 1). Several targets for vaccines have been developed. A vaccine targeting four different peptides (HLA-A*2402-restricted epitope peptides, lymphocyte antigen 6 complex locus K, TTK protein kinase, insulin-like growth factor-II mRNA-binding protein 3 and DEP domain containing 1) was explored in nine patients with refractory, advanced BTC, who were vaccinated subcutaneously once a week at doses of 0.5, 1, or 2 mg and continued until disease progression [69]. The treatment was well tolerated with no grade 3-adverse events. Peptide-specific T-cell immune responses were observed in seven patients and clinical responses were observed in six patients (66%). The median PFS and OS were 5.2 months and 12.7 months. The injection site reaction and cytotoxic T lymphocyte induction seemed to be prognostic factors for both PFS and OS.

The same team produced a vaccine targeting three HLA-A*2402 restricted epitopes: peptides-cell division cycle associated 1 (CDCA1), cadherin 3 (CDH3) and kinesin family member 20A (KIF20A) [68]. It was also well-tolerated with no grade 3–4 adverse events. All patients exhibited peptide-specific T cell immune responses. Five out of nine patients had a stable disease. Median PFS and OS were 3.4 and 9.7 months. Reaction injection site appeared to be prognostic of OS.

A randomized phase 2 study tested whether low-dose cyclophosphamide improved antigen-specific immune responses and clinical efficacy of personalized peptide vaccination in 49 advanced, pre-treated BTC patients [70]. Median PFS was significantly higher in the doublet arm (median time: 6.1 vs 2.9 months) as well as median OS (median time: 12.1 vs 5.9 months).

Other vaccine targets have been explored such as Wilm’s tumor-1 (WT1) [67] and mucin-1 (MUC1) proteins [66], with good tolerance but poor clinical responses.

Overall, vaccines targeting several antigenic peptides seem more active in BTCs that mono-peptide ones. They appear well-tolerated but results are still limited. The best targets, which probably differ according to BTC subtypes, and the optimization of adjuvant agents need to be defined.

#### 3.3.2. Cellular Therapies

Cellular therapies such as chimeric antigen receptor (CAR)-engineered T cells have also been developed in phase 1/2 studies in advanced BTCs (Table 1).

A phase 1 trial evaluated the safety, feasibility, and activity of CAR-T cell therapy in HER2-overexpressing advanced BTCs (and also in pancreatic cancers) [71]. Nine patients with BTC were included (four intrahepatic cholangiocarcinomas, four extrahepatic cholangiocarcinomas, and one gallbladder carcinoma). One patient with poorly differentiated perihilar cholangiocarcinoma obtained a good partial response for 4.5 months and 3 exhibited stable disease (of less than 5.0 months).

CAR-T cell therapy was also explored in EGFR-overexpressing advanced BTCs with 19 included patients [77]. Overall, CAR-T-EGFR cell infusion was correctly tolerated, except three patients with grade ≥3 acute fever/chills and several grade ≥3 events of lymphopenia and thrombocytopenia. Seventeen patients were evaluable, with one complete remission response and 10 stable diseases. Median PFS was 4 months (range, 2.5–22 months). More data and studies are needed regarding cellular therapies in BTCs, since results are still limited. Tolerability seems nevertheless correct.

## 4. The Future of Immune Therapies in Biliary Tract Cancers

### 4.1. Identification of Predictive Markers of Response

Current benefits of immunotherapy in BTCs are still limited to a small subset of patients. Several steps will be required to establish immunotherapy as a standard of care in BTCs. First of all, it is necessary to establish the immune and cellular mechanisms specific to BTCs that can lead to the primary response to immunotherapy. The recognition of a cancer antigen is usually considered as a key step in the initiation of the response to immunotherapy [47,78].

#### 4.1.1. Tumor Mutational Burden

Genomic instability provides one mechanism for creating unique antigenicity for a cancer cell. Consequently, tumors with high TMB respond favorably to immune checkpoint inhibitors. In the KEYNOTE-158 study, ORR were significantly improved in patients with a TMB >10 mutations per megabase (Mut/Mb) compared to those with a lower TMB [79]. Unfortunately, data for TMB in BTCs is limited. In a series of 309 patients with BTC, TMB ≥ 6 Mut/Mb accounted for 19.4% of cases whereas TMB > 20 Mut/Mb was found in only 2.9% of cases [80]. In the KEYNOTE-158 trial, none of the 63 BTC patients had a TMB > 10 Mut/Mb [79]. Of note, TMB is significantly higher in extrahepatic cholangiocarcinomas (18%) and gallbladder carcinomas (22%) in comparison with intrahepatic cholangiocarcinomas (13%) [81]. Nevertheless, in a recent retrospective study aiming at examining the performance of a universal definition of high TMB in an independent cohort of patients with solid tumors treated with immune checkpoint inhibitors, of 57 patients with hepatobiliary tumors (without distinction between BTC and hepatocellular carcinoma), four (8%) had a high TMB ≥10 Mut/Mb; none responded to immunotherapy [82]. On the contrary, six patients (12%) responded in the low TMB group. This does not mean that BTCs are precluded from the list of cancers that may benefit from immunotherapy, but it is probably crucial to develop strategies increasing antigenic presentation in BTCs such as combination of immune checkpoint inhibitors with chemotherapy or radiotherapy. The role of TMB as a biomarker of response to immune therapies is also still to be specified, as well as the optimal cut-off for TMB.

#### 4.1.2. Human Endogenous Retroviruses

Genomic instability is not the only source of cancer antigens in tumors. Clear-cell renal cell carcinoma and a subset of prostate cancers have a high prevalence of human endogenous retroviruses (HERv) [83]. HERv integrated into the human genome are largely silenced in normal cells but can become dysregulated and re-expressed in cancer and may serve as the tumor antigen signal, a potential internal trigger to sensitize tumor cells to immunotherapies. The role of HERv as a potential target in BTCs should be evaluated, but they could be used as targets for monoclonal antibody therapy, or as chimeric antigen receptor T-cell therapy or as vaccination targets [84]. Interestingly, DNA hypomethylating agents (that can increase HERv transcription, leading to increased antigen expression and up-regulation of CTLA-4 and PD-L1) and immunotherapy combinatory treatments are being tested in a phase I trial (ClinicalTrials.gov Identifier: NCT03257761), studying the combination of guadecitabine and durvalumab in patients with advanced liver, pancreatic, or biliary tract cancers.

#### 4.1.3. Synthetic Immune Responses

Not all cancers may present appropriately immunogenic antigens that endogenous T cells can effectively recognize. In those cases, new strategies of synthetic immune responses should be developed. Synthetic immune responses are the result of therapeutics that artificially bind T cells to cancer cells based on their cognate binding of a T-cell receptor to a specific-MHC complex. Examples include engineered CAR-T cells and CD3 bispecific antibody approaches [85]. A better understanding of the potential targets and a personalized approach that incorporates target expression level may be required for maximizing benefit.

#### 4.1.4. Identification of the Organ-Specific Immune Contexture

It is also important to identify the organ-specific immune contexture and to develop preclinical models specific to BTCs. Indeed, the liver is well known to provide numerous mechanisms of immunotolerance [86,87]. Myeloid-derived suppressor cells, Kupffer cells and dendritic cells promote an immunosuppressive network limiting the activation of CD8 and CD4 T cells. For instance, melanoma or lung cancer with liver metastases have reduced tumor responses, shorter PFS and a worse prognosis compared to patients without liver metastasis [88]. The liver is also a richly vascularized organ with numerous myeloid and stellate cells that make up the liver architecture. These cells, associated with cancer fibroblasts, may limit the activity of T lymphocytes [89]. The role of antiangiogenic agents in liver tumors has been demonstrated in hepatocellular carcinomas [90]. The identification of the immune contexture of BTCs would probably allow a better personalization of immunotherapy in BTC patients.

#### 4.1.5. PD-L1 Expression

In parallel with the development of specific models of BTCs, reliable and predictive biomarkers should also be developed. PD-L1 expression does not seem particularly accurate in BTCs. In the KEYNOTE-158 study, 58% of patients had PD-L1 expression >1% while only 6% of patients had a response [57]. In the PD-L1-expressing and PD-L1 non-expressing subgroups, ORR was 6.6% (4/61) and 2.9% (1/34) and median PFS was 1.9 and 2.1 months, respectively.

#### 4.1.6. DNA Damage Repair

Another exploratory strategy in BTCs could be the use of mutations in DNA damage repair (DDR) genes (germline or somatic) [91]. Indeed, cells are constantly exposed to DNA damage including base modifications, single- and double-strand breaks, base-free sites and DNA cross-links. There are several DDR pathways such as base excision repair (BER), mismatch repair (MMR), nucleotide excision repair (NER), homologous recombination (HR) and non-homologous end-joining (NHEJ). If a repair pathway is deficient, alternative repair mechanisms can be activated instead.

The tumorigenesis of BTC has been associated to DNA damage, due to inflammatory cytokines and nitric oxide-dependent mechanisms [92]. Some types of DDR deficiency are found in up to 25% of patients with BTC [91]. This is of importance since alterations in DDR genes can increase sensitivity to anti-cancer chemotherapy and radiation treatments.

Since DDR defects induce genomic instability, increasing tumor immunogenicity, a sensitization to immune therapies could be obtained by inducing DNA damage (with chemotherapy or radiotherapy), by inhibiting DDR pathways or directly by using immune therapies in patients with a known DDR deficiency.

#### 4.1.7. Microsatellite Instable Tumors

MSI tumors are tumors in which the DNA mismatch repair pathway is defective (dMMR). They arise from germline mutations in MMR genes (i.e., MLH1, MSH2, MSH6 or PMS2), called Lynch syndrome, or following silencing of the MLH1 promoter by hypermethylation, mostly due to aging. In MSI tumors, as mentioned above, neoepitopes are created that favor efficient immune responses. Patients with MSI tumors are good candidates for immune therapies [52]. Data from the KEYNOTE-158 phase II study of pembrolizumab in patients with advanced, pre-treated, non-colorectal MSI/dMMR tumors has been reported [93]. Of 22 patients with BTC, 2 had a complete response and 7 a partial response (ORR 40.9%). Median PFS was 4.2 months (95%CI: 2.1-not reached) and median OS of 24.3 months (95%CI: 6.5-not reached).

Despite being a reliable biomarker with strong evidence, MSI phenotype is rare (less than 2%) in BTC [94]. Moreover, not all MSI patients respond to immune therapies. More data is then needed to evaluate immune therapies in patients with MSI BTC, especially whether a combination (anti PD-1 and anti-CTLA4) could be more effective in these patients.

#### 4.1.8. Genetic Alterations

As previously said, BTCs are known to harbor one of the highest frequencies of targetable molecular alterations across cancer types, including *FGFR2* fusions or *IDH1/2* mutations. Recently, some genetic alterations (*BRAF, BRCA2, RNF43, TP53*) were found to be statistically associated with PD-L1 expression in BTCs [95]. Moreover, FGFR inhibitors could also modulate tumor microenvironment [96]. Interestingly, a recent study evaluated the co-occurrence of NTRK gene fusions with other therapy molecular markers in cancer patients and found an increased frequency of TMB high ≥20 Mut/Mb and MSI-H in tumors with *NTRK* fusion [97]. Whether this is confirmed in BTC is unknown. In the future, therapeutic trials should evaluate the combination of FGFR inhibitors or other targeted therapies with immune checkpoint inhibitors. More data is needed to fully understand the role of genetic alterations as predictive biomarkers of response to immune therapies in BTC.

### 4.2. Ongoing Trials

The limited response to immune checkpoint inhibitor monotherapy in unselected patients with advanced BTC emphasizes the need for biomarkers (to identify patients likely to respond) and treatment combinations (to overcome limited antitumor responses).

Several phase 2 and 3 trials are ongoing evaluating immune checkpoint inhibitors alone or in combination in BTCs (Table 2). Immune checkpoint inhibitors targeting PD-1, PD-L1 or CTLA-4 are combined with each other or with cytotoxic chemotherapy, radiotherapy of ablative therapies. Interestingly, the potential interest of combining immune checkpoint inhibitors with tyrosine kinase inhibitors is emerging in several types of cancers. In BTCs, combinations that are currently tested involve the antiangiogenic agents lenvatinib and axitinib, the MEK inhibitor cobimetinib and the PARP inhibitors olaparib and rucaparib. Novel therapies such as DKN-01 (dickkopf WNT signalling pathway inhibitor 1 (DKK1) inhibitor), sargramostim (GM-CSF) and entinostat (HDAC inhibitor) are also tested in combination with immune checkpoint inhibitors.

### 4.3. Unsolved Questions

Immune therapies remain investigational in BTCs. In fact, the trials conducted to date were small-sized and non-randomized, involved heterogeneous patient populations and suggested mild-to-moderate activity at most. Several questions remain unsolved to clarify the positioning of immune therapies in the therapeutic arsenal of BTCs:

**Is BTC type predictive of response?** Whether the type of BTC (i.e., intrahepatic versus extrahepatic cholangiocarcinoma versus gallbladder carcinoma) is predictive of response to immune therapy is currently unknown. Current and ongoing trials should include subgroup analyses in order to answering this important question.

**Are the molecular subgroups of BTC predictive of response?** Interestingly, among the four BTC subtypes described by Jusakul et al. [25], cluster 3 included intrahepatic cholangiocarcinomas which overexpress immune checkpoint genes (PD-1, PD-L2, and BTLA). These immunogenic intrahepatic cholangiocarcinomas were mutually exclusive with IDH/FGFR-driven intrahepatic cholangiocarcinomas (Cluster 4). Whether these patients would respond better to immune checkpoint inhibitors has not been tested yet.

**Could we find biomarkers to select patients that could benefit from immune therapies?** TMB is significantly higher in extrahepatic cholangiocarcinomas (18%) and gallbladder carcinomas (22%) in comparison with intrahepatic cholangiocarcinomas (13%) [81], making the former potentially more sensitive to immune checkpoint blockade. However, high TMB (i.e., >10 or >20 Mut/Mb) is rare in BTCs, and reports of efficacy of immunotherapy in these tumors are scarce and disappointing [79,80,82]. Whether PD-L1 expression is able to select the good candidates to PD1/PD-L1 inhibitors is currently poorly known [57]. As MSI, the only other potential biomarker to date for the use of immune therapies in BTC, accounts for only ~2% of patients, novel biomarkers are needed to identify BTC patients that could really benefit from immune therapies.

**Could combinations of/with immune therapies increase the sensitivity of BTCs for immune therapies?** Combinations of immune therapies between themselves or with chemotherapy, radiotherapy or targeted therapies could increase the sensitivity of BTC to immune therapies, transforming a ‘cold’ tumor into a ‘hot’ tumor. These are interesting approaches and are currently being tested (Table 2).

**What are the potential mechanisms underlying possible resistance to checkpoint inhibition?** Basically, resistance to checkpoint inhibitors can be driven by immune desert phenomenon (i.e., non-inflamed tumor with no immune infiltrate), an exhaustion of immune response (i.e., lymphocytic infiltrate has been recruited into the tumor, but has failed to clear the tumor) or an exclusion of immune response (i.e., immune cells are prevented from infiltrating the tumor via stromal and tumor cell-secretion of extracellular matrix proteins). Several mechanisms and pathways of resistance have been described [98], but not specifically in BTCs. Among those, some are tumor-intrinsic factors, such as a lack of neoantigens, epigenetic changes in cancer cells (that can alter the expression of immune-related genes), alteration of signaling pathways, and regulation of interferon-gamma pathway. Some are tumor-extrinsic factors, such as a decrease in intratumoral T cell infiltration, the presence of exosomal PD-1 (associated with the suppression of immunity against tumor), the presence of immunosuppressive cells (regulatory T cells or Tregs, or myeloid-derived suppressor cells), T cell exhaustion, epithelial-mesenchymal transition, other immune checkpoint than PD-1 and CTLA-4, indoleamine 2,3-dioxygenase (IDO, that can inhibit effector T cell functions), angiogenesis (Vascular endothelial growth factor (VEGF) also has an immunosuppressive role) and enteric microbiome (that could modulate immunity against tumor cells). Understanding these mechanisms, for instance by performing sequential biopsies before and after immune therapy, could allow novel therapeutic targets and combinations to be tested to induce immune response and overcome resistance mechanisms.

**What will be the place of immune therapies in the treatment sequence of BTC?** If some patients are identified that could benefit from immunotherapies, it will be important to precisely define the optimal sequence of treatment for these patients. Whether combinations with targeted therapies could be useful is not known. Moreover, some patients may present a targetable alteration (e.g., *FGFR2* fusion, *IDH1/2* mutation, etc.) as well as sensitivity to immune checkpoint inhibitors. For example, *NRTK* fusions seem to be more frequent in MSI and TMB-high tumors. Should one prefer immunotherapy or NTRK inhibitor in these cases? Comprehensive genomic profiling may help with precision-based therapy and allow individualization of therapeutic options for patients with advanced BTC.

## 5. Conclusions

BTCs are a heterogeneous group of tumors of poor prognosis with specific anatomic, molecular and biological features. To date, the only FDA-approved immunotherapy in BTCs is pembrolizumab for the ~2% of patients with MSI/dMMR tumors; among the vast majority of patients with MSS BTC, only a small subgroup exhibits a durable clinical benefit from checkpoint inhibitors. This subgroup is ill-defined, as currently available biomarkers such as TMB or PD-L1 expression are insufficient to correctly predict response to immune therapy in BTCs. Identification of specific biomarkers will be crucial to better select candidates to immune therapies. In the future, combinations of immune checkpoint inhibitors with chemotherapy, targeted therapies or novel types of therapy will potentially bring new treatment strategies. To date and besides MSI/dMMR tumors, immune therapies remain investigational in BTCs, and inclusion of BTC patients in clinical trials is crucial.

## Figures and Tables

**Table 1:** Selected published trials with immunotherapies in advanced biliary tract cancer.

Study	Ref	Molecule(s)	Target(s)	Phase	Patients	Population	mOS (mo)	mPFS (mo)	ORR (%)
**Immune checkpoint inhibitors in monotherapy**									
NCT02829918	[55]	Nivolumab	PD-1	2	54	2nd line and beyond	14.2	4.0	22
JapicCTI-153098	[54]	Nivolumab	PD-1	1	30	2nd line and beyond	5.2	1.4	3
KEYNOTE-028	[56,57]	Pembrolizumab	PD-1	1	24	2nd line and beyond (PDL1 > 1%)	5.7	1.8	13
KEYNOTE-158	[56,57]	Pembrolizumab	PD-1	2	104	2nd line and beyond	7.4	2.0	5.8
NCT01938612	[58]	Durvalumab	PD-L1	1	42	2nd line and beyond	8.1	2.0	4.8
NCT02699515	[59,60]	Bintrafusp alpha (M7824)	PD-L1/TGF-B	1	30	2nd line and beyond	12.7	-	23.3
NCT03833661	[61]	Bintrafusp alpha (M7824)	PD-L1/TGF-B	2	159	2nd line and beyond	-	-	10.1
**Immune checkpoint inhibitors in combination**									
NCT01938612	[58]	Durvalumab-tremelimumab	PD-L1, CTLA-4	1	65	2nd line and beyond	10.1	-	7.7
NCT03046862	[62]	Durvalumab + CISGEM	PD-L1	2	45	First-line	18.1	11.0	73.3
NCT03046862	[62]	Durvalumab-tremelimumab + CISGEM (biomarker cohort)	PD-L1, CTLA-4	2	30	First-line	15.0	13.0	50.0
NCT03046862	[62]	Durvalumab-tremelimumab + CISGEM	PD-L1, CTLA-4	2	46	First-line	20.7	11.9	73.4
JapicCTI-153098	[54]	Nivolumab + CISGEM	PD-1	2	30	First-line	15.4	4.2	36.7
CA209-538	[24]	Nivolumab + Ipilimumab	PD-1, CTLA-4	2	39	2nd line and beyond	5.7	2.9	23
NCT03796429	[63]	Toripalimab + gemcitabine-S1	PD-1	2	39	First-line	-	6.7	20.6
NCT03892577	[64]	Pembrolizumab or nivolumab + lenvatinib	PD-1, multiple TK	1	32	2nd line and beyond	11.0	4.9	25
NCT03482102	[65]	Durvalumab-tremelimumab + radiotherapy	PD-L1, CTLA-4	1	15	2nd line and beyond	-	1.8	20
**Other types of immunotherapy**									
Yamamoto et al.	[66]	One-peptide vaccine	MUC-1	1	3	2nd line and beyond	-	-	0
Kaida et al.	[67]	One-peptide vaccine	WT-1	1	16	2nd line and beyond	9.6	-	0
Aruga et al.	[68]	Three-peptide vaccine	Multiple *	1	9	2nd line and beyond	9.7	3.4	0
Aruga et al.	[69]	Four-peptide vaccine	Multiple **	1	9	2nd line and beyond	12.7	5.2	66
Shirahama et al.	[70]	Vaccine + cyclophosphamide	HLA-matched peptides	2R	25	2nd line and beyond	12.1	6.1	8.0
Shirahama et al.	[70]	Vaccine	HLA-matched peptides	2R	24	2nd line and beyond	5.9	2.9	4.2
NCT01935843	[71]	CAR-T cells	HER2	1	9	2nd line and beyond	-	-	11
NCT01869166	[71]	CAR-T cells	EGFR	1	19	2nd line and beyond	-	4.0	6 (CR)

* Peptide-cell division cycle associated 1 (CDCA1), cadherin 3 (CDH3) and kinesin family member 20A (KIF20A). ** Lymphocyte antigen 6 complex locus K, TTK protein kinase, insulin-like growth factor-II mRNA-binding protein 3 and DEP domain containing 1. CISGEM, cisplatin plus gemcitabine. CR, complete response. CTLA-4, cytotoxic T-lymphocyte–associated antigen 4. EGFR, epidermal growth factor receptor. HER2, human epidermal growth factor receptor 2. HLA, human leukocyte antigen. mo, month. mOS, median, overall survival. mPFS, median progression-free survival. ORR, overall response rate. PD-1, programmed death 1. PD-L1, programmed death ligand-1. R, randomized. TGF-B, transforming growth factor beta. TK, tyrosine kinase.

**Table 2:** Main ongoing phase 2 or 3 trials of immunotherapies in biliary tract cancer.

Molecule(s)	Target(s)	Phase	Setting	Reference
**Immune checkpoint inhibitors in monotherapy or combined**				
Pembrolizumab	PD-1	2	2nd line	NCT03110328
Pembrolizumab	PD-1	2	2nd line and beyond	NCT02628067 (KEYNOTE-158)
Pembrolizumab	PD-1	2	2nd line and beyond	NCT03695952
Nivolumab	PD-1	2	2nd line and beyond	NCT02829918
STI-3031	PD-L1	2	2nd line and beyond	NCT03999658
Bintrafusp alpha (M7824)	PD-L1, TGF-B	2	2nd line and beyond	NCT03833661
Nivolumab + ipilimumab	PD-1, CTLA-4	2	2nd line and beyond	NCT02834013
Durvalumab + tremelimumab	PD-L1, CTLA-4	2	2nd line	NCT03704480 (PRODIGE57 IMMUNOBIL)
**Immune checkpoint inhibitors plus chemotherapy**				
Pembrolizumab + CISGEM	PD-1	2	1st line and beyond	NCT03260712 (EORTC-1607ABC-09)
Pembrolizumab (or placebo) + CISGEM	PD-1	3	1st line	NCT04003636 (KEYNOTE-966)
Pembrolizumab + capecitabine-oxaliplatin	PD-1	2	2nd line and beyond	NCT03111732
Toripalimab + Gemcitabine-S1	PD-1	2	1st line	NCT03796429
Toripalimab + Gemcitabine-fluorouracil	PD-1	2	1st line	NCT03982680
Toripalimab + S1-Nab-paclitaxel	PD-1	2	1st line	NCT04027764
KN035 + gemcitabine-oxaliplatin	PD-L1	3	1st line	NCT03478488
Durvalumab (or placebo) + CISGEM	PD-L1	3	1st line	NCT03875235 (TOPAZ-1)
CISGEM ± durvalumab	PD-L1	2R	Neo-adjuvant	NCT04308174 (DEBATE)
Nivolumab + ipilimumab or CISGEM	PD-1, CTLA-4	2R	1st line and beyond	NCT03101566
Durvalumab ± tremelimumab + gemcitabine or CISGEM	PD-L1, CTLA-4	2R	1st line and beyond	NCT03473574 (AIO HEP-0117)
Durvalumab + tremelimumab + CISGEM	PD-L1, CTLA-4	2	1st line	NCT03046862
Bintrafusp alpha (or placebo) + CISGEM	PD-L1, TGF-B	3	1st line	NCT04066491 (INTR@PID)
Camrelizumab + gemcitabine-oxaliplatin	PD-1	2	1st line and beyond	NCT03486678
**Immune checkpoint inhibitors plus locoregional therapies**				
Radiotherapy + nivolumab ± ipilimumab	PD-1, CTLA-4	2R	2nd line and beyond	NCT02866383
Durvalumab + tremelimumab + radiotherapy	PD-L1, CTLA-4	2	2nd line and beyond	NCT03482102
Durvalumab + tremelimumab + TACE/RFA/ablation	PD-L1, CTLA-4	2	2nd line and beyond	NCT02821754
SIRT + durvalumab ± tremelimumab	PD-L1, CTLA-4	2R	1st line	NCT04238637 (IMMUWHY)
Camrelizumab + radiotherapy	PD-1	2	1st line	NCT03898895
**Immune checkpoint inhibitors plus other therapies**				
Atezolizumab ± cobimetinib	PD-L1, MEK	2R	2nd line and beyond	NCT03201458
CISGEM + atezolizumab ± bevacizumab	PD-L1, VEGF	2R	1st line	NCT04677504 (GO42661)
Pembrolizumab + lenvatinib	PD-1, multiple TK	2	2nd line and beyond	NCT03797326
Pembrolizumab + lenvatinib	PD-1, multiple TK	2	2nd line and beyond	NCT03895970 (LEAP-005)
Pembrolizumab + sargramostim (GM-CSF)	PD-1	2	2nd line and beyond	NCT02703714
Pembrolizumab + Peg-interferon α-2b	PD-1	2	2nd line and beyond	NCT02982720
Pembrolizumab + allogeneic natural killer cells	PD-1	2	2nd line and beyond	NCT03937895
Durvalumab + olaparib	PD-L1, PARP	2	*IDH1/2* mutation, 2nd line and beyond	NCT03991832
Durvalumab + AZD6738	PD-L1, ATR kinase	2	2nd line and beyond	NCT04298008
Nivolumab + rucaparib	PD-1	2	2nd line and beyond	NCT03639935
Nivolumab + DKN-01	PD-1, DDK1	2	2nd line and beyond	NCT04057365
Nivolumab + entinostat	PD-1, HDAC	2	2nd line and beyond	NCT03250273
Toripalimab + axitinib	PD-1, multiple TK	2	2nd line	NCT04010071
Durvalumab + guadecitabine	PD-L1; DNMTi	1b	2nd or 3rd line	NCT03257761
JS001 + lenvatinib + gemcitabine-oxaliplatin	PD-1, multiple TK	2	1st line and beyond	NCT03951597

CISGEM, cisplatin plus gemcitabine. CTLA-4, cytotoxic T-lymphocyte–associated antigen 4. DKK1, dickkopf WNT signalling pathway inhibitor 1. DNMTi: DNA methyltransferase inhibitor; GM-CSF, granulocyte-macrophage colony stimulating factor. HDAC, histone deacetylase. PARP, polyADP ribose polymerase. PD-1, programmed death 1. PD-L1, programmed death ligand-1. R, randomized. RFA, radiofrequency ablation. SIRT, selective internal radiation therapy. TACE, transarterial chemoembolization. TK, tyrosine kinase. VEGFR2, vascular endothelial growth factor receptor type 2.
